# Saving babies’ lives (SBL) – a programme to reduce neonatal mortality in rural Cambodia: study protocol for a stepped-wedge cluster-randomised trial

**DOI:** 10.1186/s12887-021-02833-7

**Published:** 2021-09-07

**Authors:** Kaajal Patel, Sopheakneary Say, Daly Leng, Manila Prak, Koung Lo, Mavuto Mukaka, Arthur Riedel, Claudia Turner

**Affiliations:** 1 Saving Babies’ Lives Programme, Angkor Hospital for Children, Tep Vong (Achamean) Road & Oum Chhay Street, Svay Dangkum, Siem Reap, Cambodia; 2 Cambodia Oxford Medical Research Unit, Angkor Hospital for Children, Tep Vong (Achamean) Road & Oum Chhay Street, Svay Dangkum, Siem Reap, Cambodia; 3 Preah Vihear Provincial Health Department, Preah Vihear, Cambodia; 4 Mahidol Oxford Tropical Medicine Research Unit, Faculty of Tropical Medicine, Mahidol University, Bangkok, 10400 Thailand; 5 Centre for Tropical Medicine and Global Health, Nuffield Department of Medicine, University of Oxford, Oxford, OX3 7FZ UK

**Keywords:** Stepped-wedge, Cluster-randomised, Cambodia, Neonatal mortality, Community health worker, Healthcare worker, Participatory learning and action, Mentorship, Health system, Implementation

## Abstract

**Background:**

Neonatal mortality remains unacceptably high. Many studies successful at reducing neonatal mortality have failed to realise similar gains at scale. Effective implementation and scale-up of interventions designed to tackle neonatal mortality is a global health priority. Multifaceted programmes targeting the continuum of neonatal care, with sustainability and scalability built into the design, can provide practical insights to solve this challenge. Cambodia has amongst the highest neonatal mortality rates in South-East Asia, with rural areas particularly affected. The primary objective of this study is the design, implementation, and assessment of the Saving Babies’ Lives programme, a package of interventions designed to reduce neonatal mortality in rural Cambodia.

**Methods:**

This study is a five-year stepped-wedge cluster-randomised trial conducted in a rural Cambodian province with an estimated annual delivery rate of 6615. The study is designed to implement and evaluate the Saving Babies’ Lives programme, which is the intervention. The Saving Babies’ Lives programme is an iterative package of neonatal interventions spanning the continuum of care and integrating into the existing health system. The Saving Babies’ Lives programme comprises two major components: participatory learning and action with community health workers, and capacity building of primary care facilities involving facility-based mentorship. Standard government service continues in control arms. Data collection covering the whole study area includes surveillance of all pregnancies, verbal and social autopsies, and quality of care surveys. Mixed methods data collection supports iteration of the complex intervention, and facilitates impact, outcome, process and economic evaluation.

**Discussion:**

Our study uses a robust study design to evaluate and develop a holistic, innovative, contextually relevant and sustainable programme that can be scaled-up to reduce neonatal mortality.

**Trial registration:**

ClinicalTrials.gov: NCT04663620. Registered on 11th December 2020, retrospectively registered.

**Supplementary Information:**

The online version contains supplementary material available at 10.1186/s12887-021-02833-7.

## Background

Neonatal mortality (death within the first 28 days of life) remains unacceptably high in many countries, amounting to 2.5 million deaths a year globally [1]. Cambodia has one of the highest neonatal mortality rates in South-East Asia [2]. Reductions in neonatal mortality lag behind those seen in childhood mortality, and neonatal deaths now account for almost half of all under five deaths [1, 3]. Acceleration of the pace of reduction of neonatal mortality is a global health priority.

Most neonatal deaths can be prevented with affordable, available interventions [3, 4], however many studies successful at reducing neonatal mortality, have failed to realise similar gains at scale [5–7]. Global progress on finding effective strategies to recast smaller-scale evidence-based programmes to large and long-term application is urgently required to reduce neonatal mortality in the low- and middle-income countries (LMICs) where it is most prevalent. Set in real-world conditions, implementation research optimises both impact and transferability of innovations, by balancing the need for pragmatism and robust evaluation methodology [8]. Iteration of intervention and implementation strategies to evolving health systems and contexts is necessary to maximise implementation success, and to succeed at scale [9].

There is no single quick fix or ‘silver bullet’ to tackle any major global health challenge [10]. To effectively reduce neonatal mortality in a sustained and scalable way, multifaceted programmes are required that target the whole neonatal period [11] and the whole neonatal healthcare system [4].

More than one-third of neonatal deaths occur on the first day of life, and three quarters within the first week [1]. However, programmes that exclusively focus on early interventions such as neonatal resuscitation training conclude that further comprehensive post-natal interventions are needed to maintain gains in neonatal survival [11]. Broad interventions embracing the entire neonatal period from birth until 28 days, and targeting all major causes of death, are called for if greater impact on neonatal health outcomes is to be achieved [4].

High-quality health systems could avert one million neonatal deaths per year globally, but LMICs, where most neonatal deaths occur, typically have weak health systems [12]. Well-functioning health systems require capacity to provide both preventative and reactive management; they must deliver both routine care for all neonates and care for sick neonates. Quality must be improved at all health system levels to achieve meaningful reductions in neonatal mortality and morbidity [4]. Combining community-based interventions and primary care strategies maximises benefits [4, 13, 14]. Further, to optimise successful implementation, programmes should be embedded and integrated into existing health services, align with government priorities, and capitalise on existing resources and guidelines [4, 15].

### Empowerment of community health workers

Many neonates die in their villages. In LMICs community health workers (CHWs) play a key role in preventive and promotive care as the frontline of formal health systems [4, 14], especially in remote villages where physical access to primary care facilities is difficult. Community mobilisation in the form of facilitated participatory learning and action (PLA) involves group discussion, problem solving and reflection, which is considered more empowering than just message giving [16–21]. PLA with women’s groups has been proven as an effective strategy to improve neonatal health outcomes, particularly in rural settings [16]. However, the role of PLA in Cambodia, with CHWs and as part of a package of interventions to improve neonatal health outcomes is less well understood [21]. PLA with CHWs can embed the intervention into the existing health system, increase community ownership, and empower the frontline health workforce [14].

### Capacity building of primary care facilities

In Cambodia 83% of women now deliver in healthcare facilities [2]. However, low-quality care has limited the potential benefits of increases in facility rather than home deliveries [22]. Poor quality care is now a bigger barrier to reducing mortality than access to care [12]. Closure of this quality gap could prevent 1.3 million neonatal deaths annually [4], and could drive further increases in health facility deliveries [23]. Training healthcare workers in LMICs can improve neonatal health outcomes [24], however there is little agreement on the most effective strategy [25]. Multifaceted initiatives that go beyond traditional course-based approaches are recommended [26, 27]. Mentorship has been shown to be effective at improving quality of care [26]. Mentorship involves collaborative teaching and learning between experienced and less-experienced healthcare workers. It is less disruptive to service provision as it is conducted in the setting of patient care. By empowering learners, in situ mentorship is an innovative and potentially sustainable strategy to improve quality of care [25, 26].

### Data

Despite LMICs carrying the vast burden of neonatal mortality, nearly all neonatal deaths are never recorded [3]. Low-quality delivery and mortality statistics in Cambodia mean neonatal epidemiology is poorly understood [28]. Understanding of epidemiology is crucial to inform effective programmes, monitor spatio-temporal trends, and measure impact of interventions.

### Study objectives

The central hypothesis is that a neonatal healthcare programme spanning the continuum of care from birth to 28 days of life, and from the community to primary care facilities, can be developed and implemented in a rural, low-resource setting with a consequent significant impact on neonatal mortality.

#### Primary objective

The design, implementation and assessment of a comprehensive, neonatal, community-based and primary healthcare programme, utilising social and medical interventions to reduce neonatal mortality in a rural Cambodian province.

#### Secondary objective

Reduce neonatal mortality in a rural Cambodian province.

#### Tertiary objectives

Describe neonatal epidemiology in a rural Cambodian province over a five-year period.

Improve community-based and primary care for all neonates in a rural Cambodian province.

- Improve neonatal healthcare provided by CHWs.
- Improve neonatal healthcare provided at primary care facilities.
- Improve linkage between health system levels.

Develop an effective neonatal programme that improves neonatal health and is contextually relevant and accepted.

- Improve neonatal health outcomes.
- Develop a feasible and acceptable programme.

Calculate cost-effectiveness of the programme and resulting changes in care, as compared to existing care provided.

## Methods and design

This study is a five-year stepped-wedge cluster-randomised trial. The intervention is the Saving Babies’ Lives (SBL) programme, which comprises two major components: participatory learning and action with CHWs, and capacity building of primary care facilities. The control is standard government service. Data collection includes surveillance of pregnancies, verbal and social autopsies for neonatal deaths and stillbirths, and quality of care surveys for CHWs and primary care facilities.

### Study setting

The study covers the whole of Preah Vihear province in North-Eastern Cambodia, which is a rural and isolated province, with a population of approximately 245,000 people, and a low population density of 17.5 people per km^2^. The mainly rural population survive principally on subsistence farming.

The government-run health system in Preah Vihear comprises 45 primary care facilities and two hospitals. A network of 578 CHWs (two per village) support the delivery of primary healthcare services. CHWs live in the villages they serve, placing them in an accessible and trusted position amongst their community [29]. The 45 primary care facilities consist of 319 healthcare workers (the vast majority nurses and midwives). The provincial health system is divided into primary care administrative groups, which include all primary care facilities, primary care workers, CHWs, villagers and villages in that geographical area.

Preah Vihear province has an estimated 6615 deliveries per year (data obtained from Preah Vihear provincial health records). An estimated 83% are facility deliveries, most of them in primary care facilities, and the remainder occur at home [2].

### Study intervention

The intervention is the SBL programme, an iterative package of interventions across the continuum of care, which is designed to integrate into the existing government-run health system. The programme design leverages our experiences in reducing neonatal mortality in hospital settings in South-East Asia [30, 31].

**Fig. 1:**
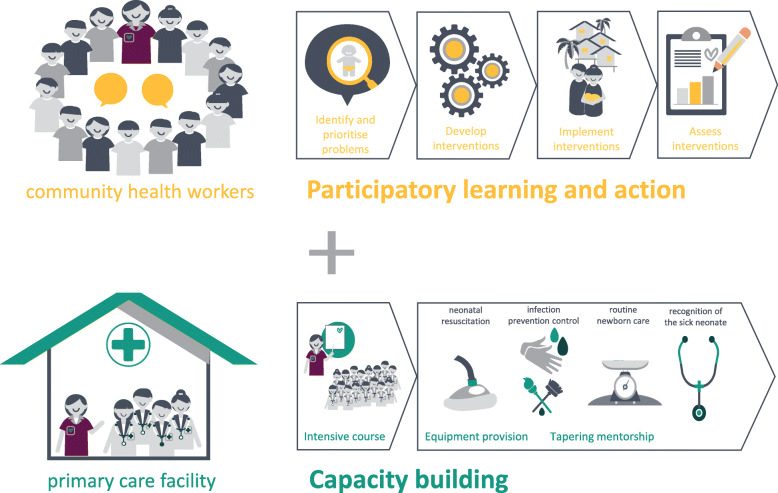
Saving Babies Lives Programme (study intervention) infographic (own image)

The SBL programme has a community-based intervention, comprising PLA with CHWs, and a primary care facility-based intervention comprising capacity building (Fig. 1). Thus, our intervention targets both demand and supply of neonatal health services. By targeting CHWs, our community intervention can reach all villages where pregnant women and neonates live, regardless of place of delivery. By targeting primary care facilities, our primary care intervention can reach most neonates, as most deliveries occur here.

The SBL programme content is based on national guidelines, where available, and was developed and is implemented in collaboration with the Cambodian Ministry of Health. The programme is implemented by Cambodian paediatric nurses in the local language (Khmer). Prior to implementation, the study team is trained in the necessary skills to implement a high-quality programme. This internal preparatory training is a part of the study intervention and will be included in the SBL programme blueprint, which will be developed for replication and scale-up.

#### Community health workers: participatory learning and action

Two years of monthly PLA sessions will be held with CHWs. The maximum PLA group size is 20. Attendance and meeting notes from all sessions are recorded. Debrief sessions are held after each meeting, and monthly facilitator meetings with study investigators permit support, discussion and documentation of key themes.

Through PLA, we provide a platform to engage CHWs in neonatal issues, and empower them to identify and address neonatal problems in their own communities. The trained study team comprise at least one facilitator and one note-keeper. They use bespoke topic guides and PLA methodology to facilitate meetings. PLA has four phases:

1. Identify and prioritise problems related to neonatal healthcare
2. Develop interventions to improve neonatal healthcare
3. Implement these interventions
4. Assess the group’s perceptions of the effectiveness of interventions

#### Primary care facility: capacity building

The primary care facility intervention has three phases:

##### Course

A three-day classroom-based course involving lectures, group discussions, and skills training. The intensive course provides the essential knowledge and skills required to provide primary level neonatal healthcare.

##### Equipment

Every primary care facility is provided with basic neonatal equipment, including a resuscitation kit, weighing scale and transfer box. Each facility assumes responsibility for maintenance and replacement thereafter. In facilities with high numbers of deliveries and adequate infrastructure, a radiant warmer is also provided.

##### Mentorship

Mentorship involves mentors assessing patients side by side with existing healthcare workers, simulation-based training and case discussions with healthcare workers. Mentorship is directed at competence in four goals: routine care of the newborn; infection prevention and control; neonatal resuscitation; and recognition of the sick neonate. Mentors are responsible for enabling each facility to achieve and sustain the four goals, by supporting healthcare workers to convert knowledge and skills acquired during the course into sustained improvements in facility practice. To achieve the goals, SBL mentors work closely with facility leaders. To encourage sustainability, mentors support problem solving of barriers to neonatal care, such as restocking systems and cleaning schedules. Additionally, two healthcare workers in each facility are trained to provide ongoing refresher training for their facility (“training of trainers”).

The mentorship has three phases, each 6 months duration, with a planned reduction in facility visits by the SBL mentor: initially 1 week per month, followed by 1 day per month, and finally 1 day every 2 months. Between visits, mentors provide remote support using telephone calls and social messaging applications. Attendance and mentor activity logs from all sessions are recorded.

An 18-month facility-based tapering mentorship involves trained SBL mentors visiting primary care facilities. Mentors are Cambodian paediatric nurses from Angkor Hospital for Children. Mentor training involves a two-day course, followed by field training, which involves accompanying experienced mentors on mentorship visits. Mentors are supported by annual refresher training and regular meetings with study investigators (Cambodian and British paediatric doctors), during which mentors for all facilities discuss progress and challenges. The study team comprises 3–5 mentors (depending on timeline), one for each facility. Mentors are also responsible for implementation of all other components of the primary care facility intervention.

### Study control

Primary care facilities (and their affiliated CHWs and healthcare workers) in control arms receive no intervention; standard government service continues.

### Study design

This study is a five-year stepped-wedge cluster-randomised trial (SW-CRT) with two arms: the SBL programme intervention and a control. To minimise contamination, clusters are based on the existing government health system structure. A cluster is defined as a primary care administrative group, as recognised by the Preah Vihear provincial health department, and includes all primary care facilities, primary care workers, CHWs, villagers and villages in that geographical area. In practice, several small facilities, serving a small population, work as a team (for example, sharing of staff). In these contexts of close association, facilities have been included in one cluster. Changes in primary care facility status, such as newly opened or upgraded facilities, which result in new clusters, are managed according to government definitions. Any changes in clusters will be reported.

**Fig. 2:**
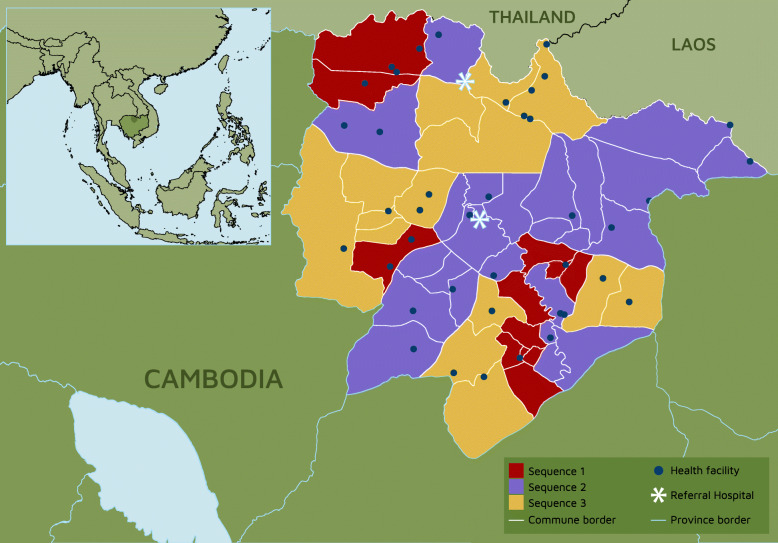
Map of Preah Vihear province, Cambodia showing clusters and health facilities. (own map points; base map data from United Nations Office for the Coordination of Humanitarian Affairs (OCHA), licensed under a Creative Commons Attribution 4.0 International License)

**Fig. 3:**
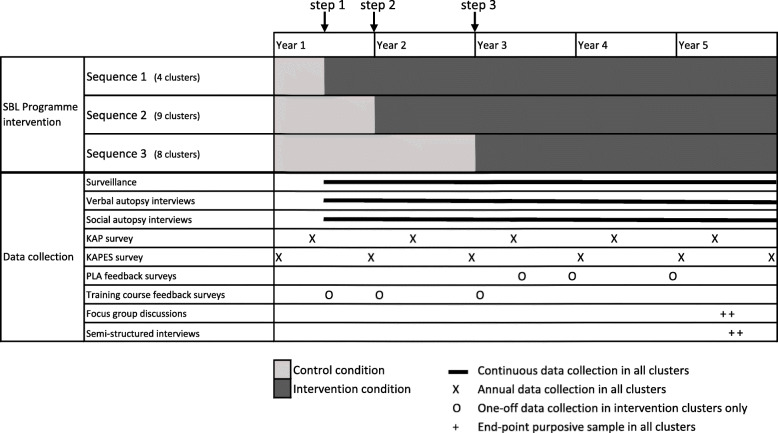
Gantt chart of stepped-wedge study design and data collection. SBL, Saving Babies’ Lives; KAP, knowledge, attitudes, practice; KAPES, knowledge, attitudes, practice, equipment, staffing; PLA, participatory learning and action

The study area (Preah Vihear province) is divided into 21 clusters (Fig. 2). Clusters were pre-assigned to one of three sequences for crossover from the control to intervention arm (Fig. 3) using covariate constrained randomisation to balance key cluster-level characteristics, including number of villages served and distance to referral hospital. The first sequence has only four clusters, to incorporate a pilot phase into study design. Appropriate services and authorities in clusters are informed about their crossover as the date approaches.

A SW-CRT design was chosen as a pragmatic study design that allows robust evaluation, whilst reconciling ethical constraints by ensuring the intervention, which is likely to be beneficial, reaches all neonates in the province. Additionally, a key concept is the iterative programme design with the intention of creating a scalable blueprint; implementation over three steps allows iteration of the SBL programme ready for wider replication. After the pilot, only minor fine-tuning of the SBL programme is expected and will be reported.

Study eligibility has both spatial and individual criteria: area is restricted to geographic clusters (Fig. 2), and the catchment population are all villagers and neonates living in this area. Study participants are all CHWs and primary care workers affiliated to all government-run primary care facilities located in the cluster.

There is a risk of contamination of the intervention in control clusters waiting for crossover because families can move freely across cluster boundaries to receive healthcare. CHWs serve the village they live in so families will likely only interact with the CHWs in their own village, and the risk of contamination of the PLA intervention is limited. However, the risk of contamination for the primary care intervention is greater: families may attend a primary care facility in another cluster, if it is located nearer to their home, for example. Possible contamination effects will be examined and reported.

### Patient and public involvement

The Cambodian Ministry of Health and policy-makers were involved in study design. Prior to study start, the study was introduced at meetings with all major stakeholder groups. Given the participatory nature of interventions, the public are involved throughout. Dissemination of study updates is ongoing via a variety of platforms locally, nationally and internationally. An open access web browser platform [32] provides real-time data and activity updates, and links to biannual reports, videos and other dissemination materials.

### Data collection

The intervention (the SBL programme) takes 2 years to implement. Anticipated effects are expected to be enduring but will take time to be realised. Consequently, observations are collected at strategic time points after intervention roll out (Fig. 3), and temporal effects, such as lag and decay, will be examined. Most observations are collected from all clusters (intervention and control groups) throughout the study (Fig. 3). If deemed necessary, data collection maybe extended for up to 1 year. Outcome measures include both cluster-level and participant-level qualitative and quantitative data. Measurements over time are an open cohort design. Observations collected under the control condition will be assessed for contamination by the intervention. Outcome measures are pre-specified in Table 1. Interim analyses inform iterations, and all data will contribute to evaluation of the intervention.

**Table 1:** Outcomes and data collection methods for evaluation of the Saving Babies’ Lives programme intervention in Preah Vihear province, Cambodia

Objectives	Expected Outcomes	Measures	Methods of data collection	Time point
*Primary objective*				
Design, implementation and assessment of an effective programme to reduce neonatal mortality	An effective programme blueprint	Replication of the SBL programme	Government records Research records	End
*Secondary objective*				
Reduce neonatal mortality	Reduction in neonatal mortality	Neonatal mortality rate	Surveillance	Continuous
*Tertiary objectives*				
Describe neonatal epidemiology over a five-year period	Description of neonatal epidemiology	Characteristics of all neonates born: gestation, birth weight, gender, birth location, season of birth	Surveillance	Continuous
		Characteristics of all neonates that die: Causes of stillbirths and neonatal deaths, contributing factors to stillbirths and neonatal deaths, location, timing, season	Surveillance, Verbal autopsy, Social autopsy	Continuous
Improve community-based and primary care for all neonates	Effective neonatal care provided by CHWs	CHW engagement: PLA attendance rate and frequency, awareness and motivation regarding neonatal healthcare, interaction between group and facilitator	PLA records	Continuous
		CHW empowerment: knowledge, attitudes and practice regarding neonatal care, number of interventions implemented by CHWs	KAP survey PLA records	Annual Continuous
	Effective neonatal care provided by primary care facilities	Facility health outcomes: facility neonatal mortality	Surveillance	Continuous
		Facility capacity to provide neonatal care: equipped and staffed facilities, skilled healthcare workers: knowledge, attitudes and practice regarding neonatal care	KAPES survey	Annual
	Effective linkage between health system levels	Neonatal healthcare linkage: referral patterns, home delivery rate, care-seeking patterns	Facility records, Surveillance, Social autopsy	Continuous
Develop an effective neonatal programme	Improved neonatal health outcomes	Perinatal mortality (stillbirth rate + death within first 7 days of live birth)	Surveillance	Continuous
		First day mortality (death within first 24 h of live birth)	Surveillance	Continuous
		Change in patterns of neonatal death: causes of death (cause-specific mortality fraction), location, timing, season	Verbal autopsy	Continuous
	Feasible and acceptable programme	CHW perceptions about programme: satisfaction, facilitators and barriers, successes and challenges	Feedback surveys, FGDs & SSIs	End
		Healthcare worker perceptions about programme: satisfaction, facilitators and barriers, successes and challenges	Feedback surveys, FGDs & SSIs	End
		Policy-maker and implementer perceptions about programme: satisfaction, facilitators and barriers, successes and challenges	Feedback surveys, FGDs & SSIs	End
		Influence or incorporation of programme components into Ministry of Health policy or strategy	Government records	End
Calculate cost-effectiveness of the intervention	Cost-effective programme	Cost-effectiveness of the intervention compared to normal standard of care: disability-adjusted life years, quality-adjusted life years	Research administrative records	End

*SBL* Saving Babies’ Lives, *CHW* Community health worker, *PLA* Participatory learning and action, *KAP* Knowledge, attitudes, practice, *FGDs* Focus group discussions, *SSIs* Semi-structured interviews, *KAPES* Knowledge, attitudes, practice, equipment, staffing

#### Health outcomes

Data collection of health outcomes is at the individual level. All pregnant women ≥28 weeks gestation living in Preah Vihear province will be recruited for the entire study period. Data collected include characteristics of all births and deaths, and the causes and social dimensions of stillbirths and neonatal deaths.

##### Neonatal mortality surveillance

To accurately determine the neonatal mortality rate (NMR) in Preah Vihear, CHWs collect data from their village on all pregnancies ≥28 weeks gestation. All pregnancies are followed up by CHWs until 28 days of life. Data on stillbirths (fetal death ≥28 weeks gestation) and neonatal survival to 28 days are collected. Data are entered into a mobile application by the study team at monthly CHW meetings held in each cluster. All primary care facility and hospital records in the province are checked regularly for stillbirths and neonatal deaths to account for deaths potentially missed and to validate data collected by CHWs.

##### Verbal autopsy

To determine the medical cause of death for all stillbirths and neonatal deaths, a verbal autopsy is performed within 6 months of death, using an adapted WHO verbal autopsy tool [33, 34]. Verbal autopsies are analysed by dual physician analysis and cause of death assigned using the new WHO classification system of ICD-10 for deaths during the perinatal period (ICD-PM) [35, 36]. Characteristics of stillbirths and neonatal deaths will also be discerned from verbal autopsy data.

##### Social autopsy

Social autopsies, conducted at the same time as verbal autopsies, collect data on contributory factors to stillbirths and neonatal deaths, such as social and demographic factors, and delays to seeking healthcare, structured according to the three-delays framework [37–40].

#### Additional outcomes

##### CHW Engagement & Empowerment

Quantitative and qualitative approaches are essential to evaluate PLA [16, 41]. Factors that will be explored include attendance, participant engagement, group cohesiveness, conveyance of messages back to their village, CHWs’ perceptions of local neonatal health issues, CHWs’ chosen interventions to improve neonatal care and reasons behind them, and facilitators and barriers faced during implementation. We also developed a knowledge, attitudes and practice (KAP) survey related to neonatal healthcare for all CHWs in the province. The KAP survey method provides a standardised and comprehensive measure of quality [42]. The KAP survey is conducted annually with CHWs entering answers directly into a mobile application.

##### Primary care facility quality

Primary care facility quality is measured annually using a quantitative and qualitative survey, similar to the CHW KAP survey. With the addition of two further domains to KAP, Equipment and Staffing, we developed a “KAPES” model of assessment for primary care facilities, based on national neonatal guidelines. All primary care workers in the province answer the knowledge and attitudes questions directly into the mobile application. The practice, equipment and staffing components are answered by the study team during annual visits to every primary care facility.

#### Qualitative evaluation

A comprehensive qualitative evaluation will contextualise quantitative results to improve SBL programme design and assess impact. Feedback surveys will be conducted with all participants of the programme. Subsequently, we will further examine ideas with focus group discussion (FGD) and semi-structured interview (SSI) methodologies. Purposive sampling will be used to select CHWs, primary care workers and health leaders across the province, to explore the acceptability and feasibility, as well as perceived facilitators and barriers, and successes and failures of the intervention. Triangulation of data from multiple sources will increase trustworthiness of findings.

#### Process evaluation

The five-year SBL study involves a complex intervention, with multifaceted components, complex interactions between interventions and outcomes, and an evolving context. Randomised controlled trials of complex interventions focussing only on outcomes are limited as they lack explanation of why the intervention worked or not. A complementary process evaluation examining implementation, mechanisms of impact, and context will help explain the main findings of our trial, and increase understanding of how to translate the SBL programme intervention from research into practice [43, 44]. We will examine interactions between intervention components and context, identify factors associated with variation in outcomes, and thus, provide insights for replication. All activities, participant attendance, decisions, challenges faced, implementer training and adaptations to the SBL programme will be examined (additional file 1) [9, 43, 44].

#### Economic evaluation

A detailed cost analysis will be carried out to estimate additional resources that are needed for the SBL programme over those required for standard care. These costs will be combined with estimates for the incremental cost of caring for neonates at facilities if attendance is found to increase, and by modelling subsequent survival benefits in terms of incremental disability-adjusted life years averted and quality-adjusted life years gained. Cost-effectiveness of the SBL programme compared to the normal standard of care (control arm) will be assessed.

### Sample size and power calculation

The recorded NMR for Cambodia was 18.4 per 1000 live births (interquartile range: 10.7–28.9) [2]. The specific NMR for Preah Vihear province is not available. The NMR in rural Cambodia is higher than in urban areas [2]. Preah Vihear is predominantly rural, so for the purposes of the sample size calculation an NMR of 28.9 per 1000 live births was used.

It is reasonable to propose that the SBL intervention will cause a one-third reduction in the NMR [4, 20], from approximately 29 per 1000 to around 19 per 1000 live births. Considering the stepped-wedge design with three steps, total 21 clusters and using an intracluster correlation coefficient (ICC) of 0.05, this study has approximately 80% power to detect a one-third drop in NMR due to the intervention. A total of approximately 26,500 (i.e. 26,460) neonates will be required. A two-sided alpha of 0.05 was used in sample size and power calculations. An ICC of 0.05 used for power calculations in the stepped-wedge design maximizes the sample size [45].

### Data analysis

Analysis of SBL programme effectiveness will be based on comparison of intervention and control groups according to the SW-CRT design and will allow for clustering and the confounding effects of time. Descriptive analysis and logistic regression will be used to compare intervention and control groups, and time points. NMR will be described by hazard rate and 95% confidence interval separated by cluster groups. Survival between groups will also be described using Kaplan-Meier plots. Log-rank test will be used for univariate comparison of survival. Epidemiological analysis will be performed on datasets from the surveillance system, verbal autopsy and social autopsy. Qualitative data from FGDs and SSIs will be analysed by content thematic analysis, and an analytical framework will be developed to identify common and emerging themes.

All analysis will be performed using the R software package [45, 46] or an alternative software. The main strategy of analysis for the NMR outcome will be according to the intention-to-treat (ITT) principle. Per protocol analyses will also be performed as a form of sensitivity analysis to the assumption of the ITT approach. Recognised risks of SW-CRTs, including methodological challenges and biases of temporal effects, intra-cluster contamination, and non-blinding of clusters, will be examined and reported [47, 48]. The final study report will follow the new Consolidated Standards of Reporting Trials extension for SW-CRTs [47].

### Data management

Ethical committee procedures on data storage, data handling and confidentiality are complied with. Following data entry, files are kept on a secure, password-encrypted server that is backed up daily. Prior to commencement, and throughout the study, the study team are trained in data management, as well as teaching and facilitation.

The SBL programme is a low risk intervention. Data checking and quality control is conducted regularly and will be reported. Study monitoring is provided by our Clinical Trials Support Group.

### Ethical considerations

Ethical considerations may emerge from PLA, depending on the interventions CHWs choose to implement. During the PLA intervention (phase 2), facilitators stimulate discussion amongst CHWs about sustainability issues related to their suggested interventions. The primary care facility intervention strategy is to implement existing national neonatal care guidelines and pathways. Consequently, no harm from participation in the primary care intervention is anticipated.

Hospitals in Cambodia lack necessary resources and skills to optimally treat sick neonates. Our SBL programme intervention will drive demand for more advanced neonatal healthcare in the province, and so ensuring availability of high quality neonatal care in referral facilities is an ethical imperative [4]. The two hospitals in the province provide services for numerous clusters (Fig. 2), and therefore we run a hospital capacity building project in parallel to the SW-CRT trial, based on a LMIC neonatal healthcare model [30, 31]. Thus, for ethical, sustainability and scalability reasons, we strengthen each level of the existing provincial health system (Additional file 2), by using a SW-CRT to implement and evaluate the SBL intervention (community and primary care) in the context of a parallel secondary care intervention, which will be reported separately.

The SBL programme involves neonatal interventions only. The importance of obstetric interventions to reduce neonatal deaths is well-recognised [4]. Unfortunately, inclusion of an obstetric component was beyond scope, as our organisation lacks the requisite obstetric expertise.

Rural populations bear a disproportionate burden of disease; risk of neonatal death in rural Cambodia is three times higher than in urban areas [2]. Rural settings hold unique challenges such as poverty, poor road infrastructure and lack of adequate health services. To achieve equity and ensure future programme scale-up reaches marginalised populations where need is often highest, we chose a particularly poor and rural province to implement the study [3, 49, 50].

The sensitive nature of a neonatal programme demands careful ethical consideration. An appropriate mourning period before approaching caregivers of the deceased is included in verbal and social autopsy methodology. Confidentiality of cases and of participant views is maintained at all times. Anonymity of data is ensured by depersonalisation at the earliest stage possible. Each participant has the right to withdraw at any time, and the reason, if given, will be recorded.

Since interventions are at cluster-level and integrate into the existing health system, the provincial government regards CHW and primary care worker participation as being part of their normal role and has requested no special consent procedures. For all individual-level data (patients, health workers), for example for neonatal mortality surveillance, verbal autopsy and KAPES surveys, informed written and/or verbal consent is taken. Both ethics committees prospectively approved all consent procedures.

The study has been approved by the Cambodian National Ethics Committee for Health Research (NECHR, 283) and the Oxford Tropical Research Ethics Committee (OxTREC, 547–17). The study is registered with clinicaltrials.gov (NCT04663620). This protocol complies with the SPIRIT checklist (Additional file 3) [51]. Important protocol modifications will be communicated with all relevant parties and will be reported.

## Discussion

With a holistic, adaptive, and innovative design, we aim to develop a contextually relevant and scalable programme that reduces neonatal mortality in LMICs. Indirect effects of the coronavirus disease 2019 (COVID-19) pandemic are having devastating impacts on gains in neonatal health, such as declines in facility deliveries and increases in neonatal deaths and stillbirths [52]. Exploring better ways to not only protect but also to accelerate improvements in neonatal health remain an urgent priority [53].

A strength of this study is the built-in sustainability and scale-up considerations for both intervention and implementation strategies. We have attempted to do so in pragmatic ways, using innovative, more resource-intense but potentially longer-lasting interventions, integration within the existing health system, stakeholder collaboration and capacity for iteration. Supporting these pillars are robust and multifaceted data collection processes, allowing mixed methods examination of intervention effects.

This study has the potential to make several important contributions to the field of neonatal global health. The first comprehensive description of neonatal epidemiology in Cambodia will support national policy-makers. Findings will contribute towards solving the challenge of sustainably reducing neonatal mortality in LMICs at scale. Furthermore, reporting of PLA amongst CHWs and of mentorship at primary care facilities can enhance understanding of the role of these innovative interventions in improving neonatal health outcomes in LMICs. We will also add to the literature through reporting of the relatively novel SW-CRT design in implementation research. If successful, our study has the potential to have far-reaching consequences to save many babies’ lives.

## Supplementary Information

**Additional file 1.** Process evaluation plan. Table showing process evaluation plan, based on a proposed framework for cluster-randomised trials of complex interventions. Participants are community health workers and primary healthcare workers.

**Additional file 2.** Infographic of whole health system strengthening (own image). Infographic of health system strengthening approach in the study province. The village (community) and health centre (primary care facility) components comprise the stepped-wedge cluster-randomised intervention. The hospital component is an additional capacity-building component implemented in parallel.

**Additional file 3.** SPIRIT checklist. Completed SPIRIT checklist including the page number from this manuscript where the issue is addressed.

## Data Availability

Data sharing is not applicable to this protocol article. The datasets used and/or analysed during this study will be available from the corresponding author on reasonable request and in accordance with organisational data sharing policy.

## Abbreviations

LMIC: Low- and middle-income countries
CHW: Community health workers
PLA: Participatory learning and action
SBL: Saving Babies’ Lives
SW-CRT: Stepped-wedge cluster-randomised trial
NMR: Neonatal mortality rate
KAP: Knowledge, attitudes and practice
KAPES: Knowledge, attitudes, practice, equipment and staffing
FGD: Focus group discussion
SSI: Semi-structured interview
ICC: Intracluster correlation coefficient
NECHR: National Ethics Committee for Health Research
OxTREC: Oxford Tropical Research Ethics Committee
