# Supplementary material for: Saving babies’ lives (SBL) – a programme to reduce neonatal mortality in rural Cambodia: study protocol for a stepped-wedge cluster-randomised trial
Source: BMC Pediatr. 2021 Sep 7;21:390. doi: 10.1186/s12887-021-02833-7 (PMC8421466; doi:10.1186/s12887-021-02833-7)
Supplement: Supplementary file 1 — Additional file 1. Process evaluation plan. Table showing process evaluation plan, based on a proposed framework for cluster-randomised trials of complex interventions. Participants are community health workers and primary healthcare workers. [file 12887_2021_2833_MOESM1_ESM.pdf]

Process evaluation plan, based on a proposed framework for cluster-randomised trials of complex interventions [1]. Participants are community health workers and primary healthcare workers.

	Domain	Research methods	Data collection source
Processes involving clusters	Cluster recruitment	Documentation of randomisation process	Research records
	Cluster reach	Comparison of clusters in each step	Government and research records
	Cluster delivery	Cluster-level intervention delivered<br>Neonates reached during mentorship	Activity reports
	Cluster fidelity	Intervention fidelity of cluster-level intervention key components and functions<br>Adaptations made to cluster-level intervention and implementation	Activity reports<br>Standard operating procedures
	Cluster response and adoption	Cluster members' perceptions of the intervention and uptake of intervention components<br>Observational and documented data about how clusters adopt the intervention	Feedback surveys<br>Meeting reports
Processes involving participants	Participant recruitment and representation	Comparison of participants receiving and not receiving the intervention	Attendance sheets
	Participant reach and selection bias	Observational and documented data about how clusters achieve reach	Activity reports
	Participant delivery	Observational and documented data about what intervention is delivered to individual participants and why	Activity reports
	Participant fidelity	Intervention fidelity of participant-level key components and functions<br>Adaptations made to participant-level intervention and implementation	Activity reports<br>Standard operating procedures
	Participant response and adoption	Observational data about participant experience of and response to the intervention<br>Analysis of what behaviour change has occurred because of the intervention	Feedback surveys<br>Team meetings
Intervention processes	Intervention sustainability	Analysis of critical processes and processes likely to be difficult to sustain	All research records
	Intervention effectiveness	Analysis of associations between trial processes and outcomes	
	Intervention unintended consequences	Data collection of potential unintended consequences (perverse, harmful or beneficial)	

1. Grant A, Treweek S, Dreischulte T, Foy R, Guthrie B. Process evaluations for cluster-randomised trials of complex interventions: a proposed framework for design and reporting. Trials. 2013;14(1):15.
