# Supplementary material for: Saving babies’ lives (SBL) – a programme to reduce neonatal mortality in rural Cambodia: study protocol for a stepped-wedge cluster-randomised trial
Source: BMC Pediatr. 2021 Sep 7;21:390. doi: 10.1186/s12887-021-02833-7 (PMC8421466; doi:10.1186/s12887-021-02833-7)
Supplement: Supplementary file 2 — Additional file 2. Infographic of whole health system strengthening (own image). Infographic of health system strengthening approach in the study province. The village (community) and health centre (primary care facility) components comprise the stepped-wedge cluster-randomised intervention. The hospital component is an additional capacity-building component implemented in parallel. [file 12887_2021_2833_MOESM2_ESM.pdf]

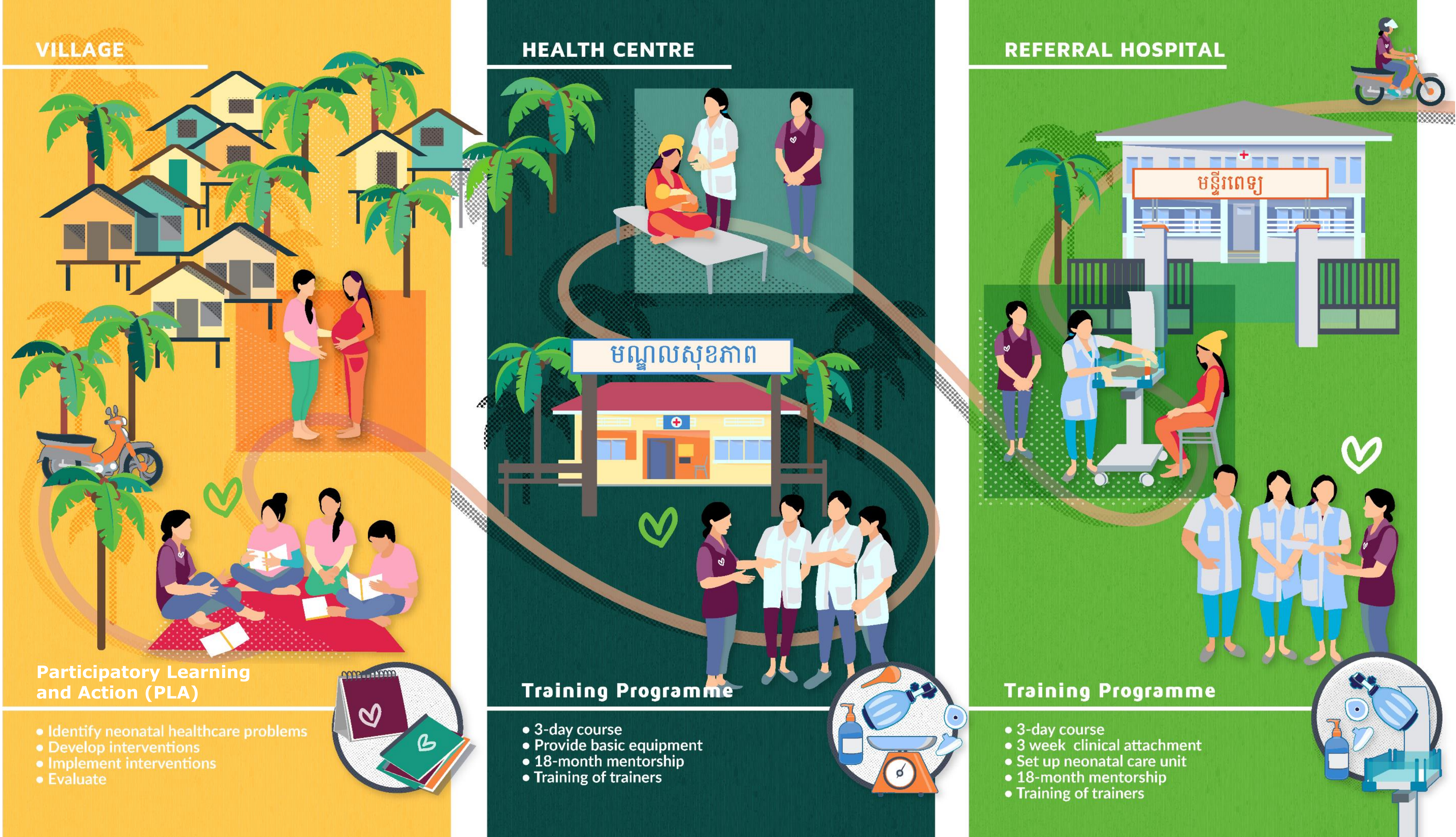
VILLAGE
Participatory Learning and Action (PLA)
• Identify neonatal healthcare problems
• Develop interventions
• Implement interventions
• Evaluate
HEALTH CENTRE
មណ្ឌលសុខភាព
Training Programme
• 3-day course
• Provide basic equipment
• 18-month mentorship
• Training of trainers
REFERRAL HOSPITAL
មន្ទីរពេទ្យ
Training Programme
• 3-day course
• 3 week clinical attachment
• Set up neonatal care unit
• 18-month mentorship
• Training of trainers
